# Complete Resection of a Torcular Herophili Hemangiopericytoma without Sinus Reconstruction: A Case Report and Review of the Literature

**DOI:** 10.1155/2023/2349363

**Published:** 2023-09-06

**Authors:** Salah-Edine Safi, Julie Godfrain, Herbert Rooijakkers, Frederic Collignon

**Affiliations:** Centre Hospitalier Interrégional Edith Cavell, Brussels, Belgium

## Abstract

A 78-year-old woman presented to the emergency department with mild headaches and a sudden onset of blurred vision. Computerized tomography scan and magnetic resonance imaging showed what was described at first as a meningioma invading and occluding the torcular Herophili, the posterior third of the superior sagittal sinus and the proximal part of the right transverse sinus. Gross total resection of the tumor was performed without reconstructing dural sinuses. The patient was discharged home without new neurological deficit. Histopathology was in favor of a hemangiopericytoma Grade II World Health Organization (WHO). Total body positron emission tomography [^18^F]fluorodeoxyglucose found no secondary location. Radiotherapy was planned post-operatively.

## 1. Introduction

Hemangiopericytomas (HPCs) are a rare mesenchymal tumor. They represent approximately 1% of all intracranial tumors are thought to originate from Zimmerman's pericytes [1] and are located in the arachnoid space similar to meningiomas. HPCs are more aggressive than meningiomas [2] with a tendency of local recurrence and extracranial metastases [1, 2]. Since 2016, HPCs have been reclassified by the WHO and are a specific entity: solitary fibrous tumors. The Grade I is a solitary fibrous tumor, Grade II is hemangiopericytoma, and Grade III corresponds to anaplastic hemangiopericytoma [3].

HPCs are more common in males and the mean age of diagnosis is lower compared with meningiomas [2]. The extent of surgical resection and post-operative radiotherapy (PORT) is correlated with overall survival and recurrence-free survival [2, 4]. Confluent of sinuses is a rare location for the development of HPCs. To the best of our knowledge, only three cases of torcular Herophili hemangiopericytoma complete resection with dural sinus reconstruction using various grafts have been described [5–7]. In our report, we describe the gross total resection (GTR) of a torcular Herophili hemangiopericytoma with venous sinus resection instead of reconstruction.

## 2. Case Presentation

**Figure 1:**
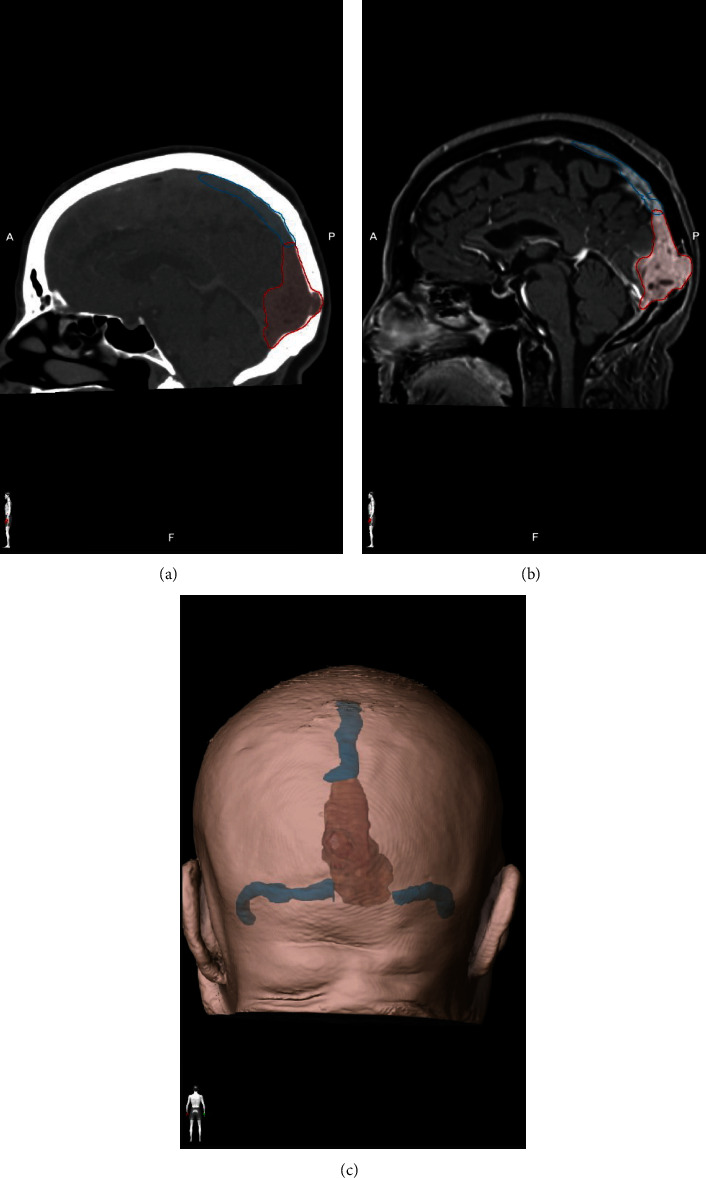
Representative images of intracranial hemangiopericytoma invading the CoS, the SSS, and RTS. (a) CT scan images shows the bony invasion. (b) MRI shows heterogeneously enhancing mass inside the confluent of sinuses. (c) Brainlab^®^ 3D reconstruction of the tumor (red) in relation with dural sinuses (blue).

**Figure 2:**
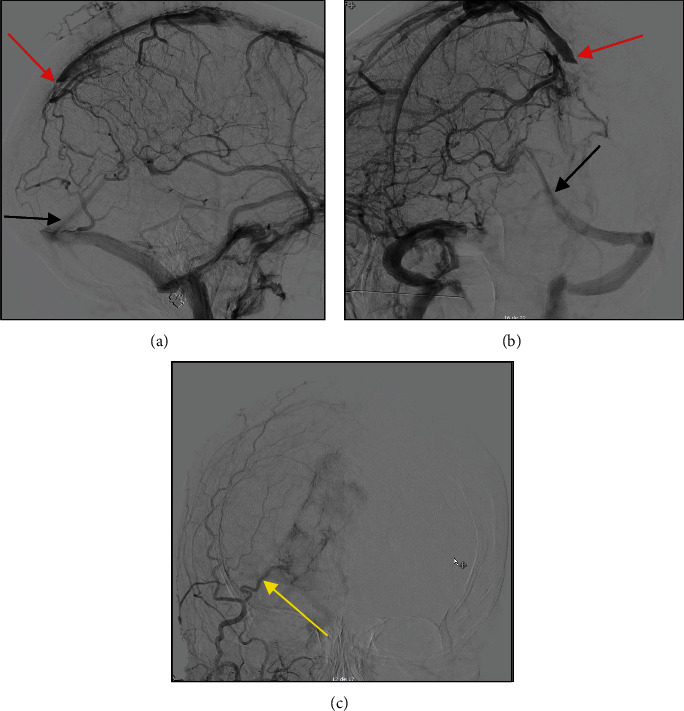
(a and b) Pre-operative carotid angiogram revealing the occlusion of the posterior third of the SSS (red arrow) by the tumor. (c) Right external carotid angiogram, moderate tumor stain feeding from the occipital artery (yellow arrow) was observed. The strait sinus directly connects to the left transverse sinus (black arrow).

A 78-year-old woman was admitted to the hospital for sudden onset of bilateral blurred vision associated with mild headaches. Neurological examination showed no other neurological deficit. The ophthalmological report found no sign of papilledema and described a loss of central visual field bilaterally. A computerized tomography (CT) scan reported a medial occipital iso-dense multi-lobulated lesion with occipital bone erosion and intense contrast enhancement. Magnetic resonance imaging (MRI) imaging showed an invasion of the posterior third of the superior sagittal sinus (SSS), the confluence of sinuses (CoS), and the proximal third of the right transverse sinus (RTS) with bone invasion (Figure 1). The radiological report concluded to a meningioma. Due to the invasive nature of the lesion, we ordered a cerebral angiogram, which confirmed a complete occlusion of the venous sinuses with efficient drainage from cortical veins to the sigmoid sinus. This could explain the lack of papilledema and increased intracranial pressure (ICP). The tumor was supplied by both occipital arteries, dural and adjacent parenchymal arteries (Figure 2). Pre-operative embolization of the occipital arteries was performed to minimize intra-operative bleeding. Due to its complexity, the case was discussed with the other team members of our neurosurgical department. The senior author, Pr Collignon, raised the possibility of the tumor being an HPC and thus a more aggressive approach could be of benefit to the patient. Therefore, a complete resection of the tumor with a reconstruction of the sinus was planned.

## 3. Surgical Procedure

**Figure 3:**
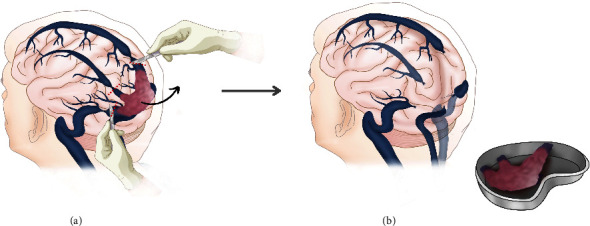
Illustration of the surgical steps. After exposing the CoS, bilateral TSs, and distal SSS. (a) 4 silk knots were closed at the edges of the tumor. (b) Complete removal of the dural sinuses after partial debulking of the tumor was performed.

**Figure 4:**
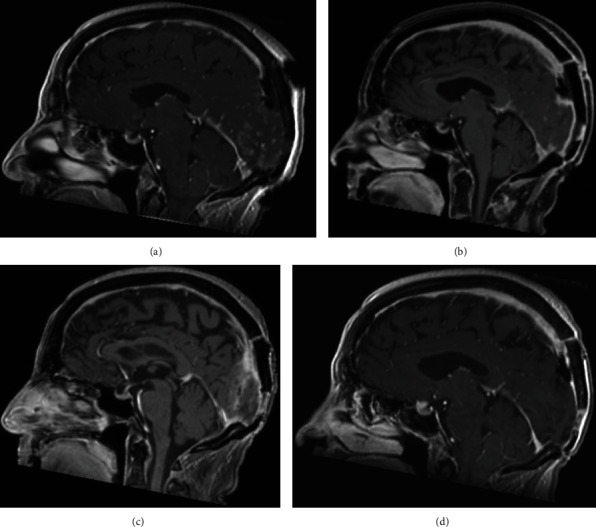
Post-operative MRI confirming the GTR of the tumor with no signs of recurrence at (a) 24 hours, (b) 3 months, (c) 6 months, and (d) 12 months. No signs of cerebral venous infarction after complete removal of the tumor.

The patient was in a prone position, head fixed in a Mayfield head clamp. A median occipital horseshoe incision was performed followed by a bilateral occipital craniotomy. The tumor was initially debulked using an ultrasonic aspirator. Heavy and diffuse bleeding was encountered despite pre-operative embolization rendering the resection very challenging. During debulking, the tumor was found to have invaded the sinus walls. The decision was then made to go for an en-bloc resection instead of a reconstruction. The sinuses were occluded with silk knots before resecting the invaded part of the SSS and RTS (Figure 3). After sinus occlusion, no brain swelling or venous infarction was encountered, which helped in the decision regarding the reconstruction of the sinuses. Dura was closed using Duragen^®^. Total operative time was 215 minutes, and total blood loss was estimated at 2 L. Two red blood cell units were required during the operation. The immediate post-operative period was marked by a worsening of the vision followed by a progressive improvement during the following days. The patient stayed 2 days in the intensive care unit and was discharged on day 7 after surgery. Post-operative MRI showed complete resection and no recurrence at 3, 6, and 12 months (Figure 4). Histopathology concluded to a Grade II hemangiopericytoma (STAT6 and CD34 positive, Ki67 at 5% with some rare hot spots at 10%, and 2 mitoses per 10 High Power Field (HPF)). Following multidisciplinary discussion, the patient received radiotherapy with a total of 60 grays delivered in 30 sessions with good tolerance.

## 4. Discussion

### 4.1. Radiological Characteristics

HPC and meningiomas share some similar radiological features with some key differences [8]. On CT scan they both present as iso-dense and after contrast injection enhance intensely. However, meningiomas tend to cause reactive hyperostosis, whereas HPCs tend to erode the bone [8].

In MRI studies, solitary fibrous tumor (SFT)/HPC have a higher diffusion coefficient, lack a dural tail, or present with a narrow-based dural attachment. They also tend to present with less peritumoral brain edema and show extensive serpentine flow void corresponding to higher vasculature. This study also showed fewer differences between angiomatous meningiomas and SFT/HPC [9].

On diffusion imaging, the normalized Apparent Diffusion Coefficient (ADC) values (nADC) and degree of intratumoral susceptibility signal intensity (ITSS) were significantly higher in intracranial hemangiopericytoma than those in meningiomas [10]. The threshold value of >1.15 for nADC provided 75.00% sensitivity and 60.42% specificity for differentiating SFT/HPC from meningiomas. Compared with nADC, the degree of ITSS had a moderate sensitivity (62.50%) and a higher specificity (85.42%) using the threshold value of >1.00.

In MR spectrography myo-inositol values superior or equal to 6,347 in short echo-time were associated with the diagnosis of HPC/SFT brain tumors with high sensitivity and specificity [3].

It has to be mentioned that the definitive diagnosis of an HPC can only be made after a histopathology study [11] but the radiological findings can give valuable information as to the possibility of the tumor being an HPC and help plan the surgery accordingly.

### 4.2. Prognosis

Patients suffering from HPC have a lower life expectancy compared to those with meningiomas because of the higher rate of recurrence and the risks of extracranial metastasis [11]. Gubian et al. showed that SFT/HPC can have very unpredictable clinical behavior. In fact, in their series of 29 patients even the ones with a diagnosis of SFT with gross-total resection (GTR) had a recurrence and for one patient a malignant transformation [12]. Rutkowski et al. [13] reported in their systematic review an overall median survival of 13 years, with 1-, 5-, 10-, and 20-year survival rates of 95%, 82%, 60%, and 23%, respectively. GTR has the greatest survival (median survival of 9.75 years) and should always be the first goal when possible. The subtotal resection followed by radiation therapy has a median survival of 6 years.

Comparatively, the 5-year survival rate for Grade I meningiomas is estimated at 91.3% [14]. For Grade II, survival rates range from 83.2% to 93.5% [15–17]. The 10-year survival rate is 83.4% [17]. The 2-, 5-, and 10-year overall survival rates were 82%, 61%, and 40%, respectively, for Grade III [18].

PORT is a debated matter in the literature. Some authors showed real benefits after GTR and partial resection with a reduction in local recurrence [19, 20]. Other studies suggest that radiotherapy does not improve local control or survival rates [21]. In our case, we opted for a PORT after a multidisciplinary discussion. The patient underwent PORT 1 month after surgery. A total of 60 grays were delivered in 30 sessions [22] as we estimated that despite her age the patient was in a very good health condition, and we wanted to improve local control. A usual dose of 50–60 Gy in 25–30 fractions can be suitable for Grade II, some even advocate for more than 60 Gy [23], hemangiopericytoma, and in cases of Grade III tumors, higher doses are needed [21].

### 4.3. Surgical Strategy

Due to the high local recurrence rate and distant metastasis of HPCs an aggressive surgery should be attempted when possible. For hemangiopericytoma invading the CoS symptom severity depends largely on the efficiency of the venous drainage. To the best of our knowledge, only three cases of CoS HPCs with dural sinus reconstruction have been reported. The first was in 1994 by Nagashima et al. [7] where they reported the case of a hemangiopericytoma invading the straight and transverse sinuses. In their preoperative investigations, they observed a collateral bypass from the SSS to the straight sinus with a lack of opacification of the Rosenthal veins. This suggested a poor deep venous drainage, and therefore, prompted a sinus reconstuction using a radial artery graft. Collignon et al. [6] described the case of a 38-year-old man with a hemangiopericytoma of the CoS and both transverse sinuses with bilateral papilledema and increased drainage by facial and both superior and inferior ophthalmic veins. These findings led them to choose a reconstruction of the transverse sinus using bovine pericardium to restore good venous drainage and reduce the ICP. The last case was described by Kurisu et al. [5]. In their case, chronic CoS obstruction led to the development of a dural Arterio-Veinous Fistula (dAVF) and increased ICP. This encouraged them to perform a complete resection of the tumor with dural sinus repair using a Gore-Tex artificial membrane. Following surgery, the dAVF spontaneously regressed.

At first, we had some confidence that the tumor was an HPC so we preoperatively planned a GTR with the reconstruction of the sinus using the pericardial patch. During surgery, because of the heavy bleeding, we opted for an en-bloc resection. Our decision was supported by several pre- and post-operative findings. First, the tumor invaded the entirety of the sinus walls, leaving no intact tissue that could be salvaged in a reconstruction. Second, the patient had a chronic and complete obstruction of the CoS, SSS, and proximal third of the RTS with no signs of increased ICP or venous infarction. Furthermore, the angiogram showed efficient venous drainage as proven by the early opacification of the sigmoid sinus after cortical venous outflows. Moreover, after occlusion of the sinuses with silk per-operatively we encountered no brain swelling or venous infarction. Additionally, we encountered no venous drainage in the bone or the scalp, which further suggests a good intracranial venous outflow. Finally, since the patient was 78 years old we wanted to prevent high morbidity that is associated with an extended surgical time due to the reconstruction of dural sinuses. Good clinical result was achieved as shown by the rapid recovery of the patient and the absence of post-operative complications.

The patient received a 60 Gy dose of radiation therapy for several reasons. Despite her age, she was in a very good health condition and only had a history of mild hypothyroidism. Moreover, since the topic is still very debated in the literature no real guidelines are established. Finally, our team wanted to prevent a local recurrence as much as possible and the patient agreed to the proposed treatment.

The use of preoperative embolization is known to reduce preoperative bleeding as well as decrease mortality and morbidity related to hemangiopericytoma surgery [10, 24]. Unfortunately, in our case, embolization had a limited role since the majority of the tumor's blood supply came from meningeal and cortical feeders. The need for preoperative embolization should be carefully assessed and only favorable cases should be selected.

## 5. Conclusion

In this report, we describe the resection of a CoS HPC without reconstruction of the SSS and the RTS. This surgical strategy is viable in very select cases where cerebral venous drainage is not compromised. We also described some of the radiological differences between HPC and meningiomas, which can be helpful in the decision-making process for the surgical strategy. A cerebral angiogram should be mandatory in cases involving dural sinuses for the assessment of arterial supply and venous drainage. Preoperative embolization can be of good use in reducing preoperative bleeding and cases that could benefit from it should be carefully selected. Finally, GTR should always be the first intention when possible and radiotherapy should be administered to reduce as much as possible the local recurrence.
